# Clinical analysis and case series report on autoimmune glial fibrillary acidic protein astrocytopathy presenting with autonomic dysfunction

**DOI:** 10.3389/fneur.2024.1464891

**Published:** 2025-01-31

**Authors:** Xiao Ke Wu, Guojie Zhai, Jin Ru Zhang, Hua Ping Du, Lan Hu, Shu Ye Yu, Hai Lun Hang, Sirui Han, Yanlin Zhang, Yuan Xu

**Affiliations:** ^1^Suzhou Ninth People's Hospital, Suzhou, China; ^2^Department of Neurology, The Second Affiliated Hospital of Soochow University, Suzhou, China; ^3^Kang da College of Nanjing Medical University, Lianyungang, China

**Keywords:** GFAP-A, autonomic dysfunction, prognosis, urinary bladder dysfunction, autoimmune glial fibrillary acidic protein astrocytopathy

## Abstract

**Background and purpose:**

The incidence of autonomic dysfunction is frequently underestimated and often overlooked in patients with autoimmune glial fibrillary acidic protein astrocytopathy (GFAP-A). This study comprehensively analyzes the clinical manifestations, laboratory and imaging findings, and treatment modalities for patients demonstrating GFAP-A involvement in the autonomic nervous system. The present article primarily elucidates the prevalence and prognosis of diverse autonomic system symptoms while examining the associated laboratory and imaging indicators. These findings aim to establish a foundation for personalized diagnosis and treatment strategies in such patients.

**Method:**

We performed a retrospective data analysis from three cases of GFAP-A involvement in autonomic nerves from the Ninth People’s Hospital of Suzhou and the Second Affiliated Hospital of Soochow University from 2020 to 2023. After an extensive literature review, we identified 39 cases involving GFAP-A and autonomic nervous system dysfunction. We also comprehensively analyzed the patient’s clinical manifestations, laboratory biomarkers, and imaging findings.

**Result:**

The study included a total of 42 patients, consisting of 15 females and 27 males. The primary manifestations of autonomic dysfunction included bladder dysfunction (41/42 cases), gastrointestinal dysfunction (18 /42 cases), heart rate variability (4/42 cases), unusual sweating (2/42 cases), and blood pressure fluctuations (1/42 cases). Other neurological symptoms comprised headache (12 /42 cases), limb weakness presentation (30/42 cases). Blood pressure variability is related to cerebrospinal fluid pressure and convulsive seizures. Heart rate variability is related to disorders of consciousness. unusual sweating was associated to age, gender, cerebrospinal fluid protein content and convulsive seizures. Gastrointestinal disorders have associations with gender, sleep disturbances, protein content in the cerebrospinal fluid, and brain MRI lesions. The prognosis of autonomic nerve dysfunction is associated to sleep disorder and cerebrospinal fluid protein content. The higher the cerebrospinal fluid protein level, the worse the prognosis of autonomic nervous system.

**Conclusion:**

Bladder dysfunction and gastrointestinal dysfunction are the primary manifestations of autonomic dysfunction in GFAP-A patients, with a subset also experiencing abnormalities in heart rate, blood pressure, and sweating. These additional symptoms have implications for prognosis, necessitating heightened attention from clinicians toward GFAP-A patients.

## 1 Introduction

Autoimmune glial fibrillary acidic protein astrocytopathy (GFAP-A)is a central nervous system disorder first reported and named by Lennon’s team in 2016 (1). To date, the full spectrum of autoimmune GFAP astrocytopathy including epidemiological, pathophysiological, clinical and radiological presentations, diagnostic criteria, treatment regimens and prognosis is still remained obscure due to the nonspecific symptoms and lack of longterm follow-up data. In 2021, a study reported the clinical, imaging, and laboratory characteristics of 18 patients with autoimmune GFAP astrocytosis. They also reviewed and summarized all 306 previously reported cases, expanding our understanding of the clinical and neurophenotypic aspects of autoimmune GFAP astrocytosis (2). In 2022, a multicenter study recruited approximately 300 GFAP-A patients from 27 hospitals in China. The study proposed a reasonable estimate of the appropriate sample size, which is the largest sample size for GFAP-A research to date and is still under investigation. We look forward to the research results (3).The specific biological antibody GFAP IgG of GFAP-A can be detected by histological and cytological methods to detect antibodies in cerebrospinal fluid, recent studies suggest that cerebrospinal fluid GFAP antibodies have high specificity and sensitivity, and have found that the positivity rate and titer of cerebrospinal fluid GFAP antibodies are significantly higher than those of serum. The specificity of serum GFAP antibodies still needs further evaluation (1). The main clinical manifestations of GFAP-A include acute or subacute episodes of steroid-responsive meningoencephalitis and myelitis or localized meningoencephalitis or myelitis, which may present as fever, headache, encephalopathy, myelitis, unusual vision, involuntary movement, ataxia, mental disorders, epilepsy. The lesions typically involve the white matter region, subcortex, thalamus, basal ganglia region, brainstem, and cerebellum. Long segments characterize most cases of myelopathy and can affect both cervical and thoracic segments (4). The characteristic MRI feature of GFAP-A is the radial gadolinium enhancement surrounding the linear cerebral blood vessels in the white matter, oriented perpendicular to the ventricles (5). The clinical phenotype of GFAP-A also includes autonomic dysfunction, which includes bladder dysfunction, gastrointestinal symptoms, heart rate variability, blood pressure variability, and unusual sweating (6). The clinical presentation of bladder dysfunction may include increased frequency of urination, urgency incontinence, and urge incontinence, as well as dysuria and overflow incontinence (7). The clinical presentation of gastrointestinal dysfunction may include symptoms such as abdominal pain, diarrhea, nausea, vomiting, belching, and anorexia (8). Heart rate variability refers to the fluctuation in the time intervals between heartbeats, serving as a sensitive indicator of autonomic nervous system regulation and cardiac homeostasis (9). Blood pressure variability helps detect circadian changes in blood pressure. It can be divided into riser blood pressure rhythm (nighttime blood pressure decrease ≥10%) or non-dipper blood pressure rhythm (<10% drop in nighttime blood pressure) based on the reduction of average systolic blood pressure at night compared to daytime (rate of nocturnal blood pressure decrease). His non-dipper blood pressure can be a result of autonomic disorders and an elevated risk of target organ damage and cardiovascular mortality (10). Patients with autonomic dysfunction are uncommon in clinical practice. However, when autonomic dysfunction appears in the early stages of GFAP-A patients’ disease course, their symptoms may be atypical and incomplete, potentially leading to clinical negligence and misdiagnosis. Hence, we conducted a descriptive analysis of the clinical symptoms, auxiliary examinations, and treatment plans for 42 patients with GFAP-A (3 cases in our hospital and 39 cases from the literature) and investigated the correlation between the incidence and prognosis of autonomic symptoms with other clinical symptoms and auxiliary examinations.

## 2 Method

### 2.1 Case information

We enrolled 5 GFAP-A patients admitted to the Ninth People’s Hospital of Suzhou and the Second Affiliated Hospital of Soochow University from 2020 to 2023, all patients tested positive for GFAP IgG in their cerebrospinal fluid. We obtain clinical data through medical records and telephone interviews. Autonomic symptoms encompass bladder and gastrointestinal system dysfunction, blood pressure and heart rate variability, and unusual sweating. The study included the last three patients presenting with autonomic symptoms, excluding two patients - one due to incomplete data and the other for not exhibiting any autonomic symptoms. The study involved the analysis of autonomic phenotypic data, including the phenotype of autonomic symptoms, incidence, and response to immunotherapy. It also encompassed demographics, other clinical symptoms, cerebrospinal fluid, and MRI features. We thoroughly reviewed the treatment and outcomes of these patients using the modified Rankin Scale (mRS) to assess the severity of the disease and follow-up outcomes.

We conducted a systematic search using PubMed and Embase with the following search terms: (GFAP astrocytopathy OR GFAP autoimmunity OR autoimmune glial fibrillary acidic protein astrocytopathy OR GFAP autoantibody), covering the time frame from January 2016 to December 2023, to identify published cases of GFAP-IgG-positive involving the autonomic nervous system. The included literature encompasses original studies reporting on one or more GFAP-A patients, as well as Chinese articles, which detail the clinical characteristics of patients with documented GFAP-IgG positivity. This includes case reports/series, cohort studies, and case–control studies. Studies that solely contain animal data, conference abstracts, and those with incomplete clinical data were excluded. We comprehensively reviewed all previously documented cases, totaling 42, with adequate clinical data. We excluded two cases with partial data missing, one due to missing demographic data and incomplete cerebrospinal fluid experimental testing data. Finally, we summarized the phenotypes of autonomic symptoms in 42 samples (including 3 cases in our study and 39 cases in existing literature).

### 2.2 Data extraction

Demographic information and disease history: age, gender, family history of diseases, comorbidities.Clinical features: history of precursor infection and pathogenic pathogen information, onset characteristics, clinical manifestations (such as fever, headache, encephalopathy, involuntary movement, myelitis, visual abnormalities, ataxia, mental disorders, epilepsy, and autonomic dysfunction), and other symptoms and signs of meningoencephalomyelitis. Laboratory tests: blood tests (blood routine, biochemical, tumor marker assessment, thyroid function markers, and rheumatic disease markers) and spinal fluid tests (biochemical, cytological, and bacteriological analysis). GFAP antibody screening test: detecting GFAP antibodies in CSF or serum through indirect immunofluorescence assay. Imaging examination, including MRI and enhancement of the head and spinal cord. EEG examination. Record immunomodulatory treatment plans, including intravenous methylprednisolone, intravenous immunoglobulin, plasma exchange, immunoadsorption, oral corticosteroids, and immunosuppressive drugs. Disease prognosis.

### 2.3 Data analysis

We used SPSS version 20.0 for statistical analysis. We presented descriptive statistics as the mean ± SD or median (range) for continuous variables and frequency and percentage for categorical variables. Using paired sample t-test and chi square test to compare the associations between different autonomic nervous system symptoms and other clinical symptoms, age, gender, positive imaging findings, and laboratory results of patients, and incorporate significant indicators into binary logistic regression to explore their independent association. We set the statistical significance level to *p* < 0.05.

## 3 Result

We identified three male patients with autonomic symptom phenotype GFAP-A, all of whom tested positive for serum GFAP IgG (Mean age: 62.66 ± 9.01 years). Serum AQP4 IgG was negative in all patients and no potential malignancies. Follow-up results showed partial improvement in autonomic nerve symptoms, with all patients scoring 1 point on the mRS Scale Table 1.

**Table 1:** Demography, autonomic symptom phenotype, score and clinical characteristics of 3 patients with glial fibrillary acidic protein IgG positive.

Case	Age(year)/ sex	Autonomic symptoms	Clinical manifestations and symptoms.	Cerebrospinal fluid pressure(mmH2O) Cell count in cerebrospinal fluid (μL) Proteins in the cerebrospinal fluid (g/L) The glucose level in the cerebrospinal fluid (mmol/L)	Autoimmune encephalitis antibody/s generation sequencing	GFAP concentration (CSF/Serum)	MRI (Head/Spinal cord)	SCOPA-AUT score	revitalize	Autonomic symptoms prognosis	Prognostic mRS Score
Case1	72/ male	Urinary incontinence Urinary retention	Weakness in the bilateral lower extremities	160 4 0.7 3	Negative/Negative	1:10/−	normal /Multiple abnormal signals indicating demyelinating changes in the cervical and thoracic spine pulp	28 points	IVMP	Partial improvement	1 point
Case2	54/ male	Urinary retention	fever Impairment of consciousness Weakness in the bilateral lower extremities	300 392 1.772 3.39	Negative/Negative	1:32/−	Multifocal flaky T2/FLAIR hyperintensity changes observed in the periventricular white matter. /The nerve root walking area on both sides of T5-T10 exhibited low signal intensity on T1-weighted imaging and high signal intensity on T2-weighted imaging.	10 points	IVMP	Partial improvement	1 point
Case3	62/ male	Urinary retention	fever headache Trembling of lower limbs	240 125 1.8 4.85	Negative/Negative	1:32/1:100	normal /normal	10 points	IVMP Gamma globulin	Partial improvement	1 point

As of December 2023, we have collected literature on 38 patients with autoimmune GFAP astrocyte disease who have reported autonomic symptoms (5, 11–23). The literature included 39 patients, with a mean age of 44.72 ± 16.54 years and 36.58% female. Our analysis revealed that the primary indications of autonomic dysfunction included bladder dysfunction (41/42cases), gastrointestinal dysfunction (18/42cases), heart rate variability (4/42 cases), anomalous sweating (2/42cases), and blood pressure variability (1/42 cases). Other neurological symptoms were headache (12/42cases), dizziness (2/42cases), disturbance of consciousness (4/42 cases), dyspnea (2/42 cases), epileptic seizure (7/42 cases), convulsive seizure (1/42 cases), visual disturbance (11/42 cases), polar posterior syndrome (6/42 cases), bulbar palsy true (3/42 cases). Paresthesia (14/42 cases), involuntary movement (3/42 cases), ataxia (4/42 cases), limb weakness (30/42 cases), paresthesia (14/42 cases), cognitive impairment (3/42 cases), psychiatric symptoms (6/42 cases), sleep disturbance (3/42 cases), Other clinical symptoms were fever (20/42 cases), rash (2/42 cases); The findings from the lumbar puncture combined with cerebrospinal fluid examination of 42 patients revealed that 27 patients exhibited elevated cerebrospinal fluid pressure, 39 patients had an increased count of cerebrospinal fluid white blood cells, 29 patients showed elevated levels of cerebrospinal fluid protein, and nine patients displayed decreased CSF sugar content; Magnetic resonance imaging (MRI) findings revealed abnormalities in the head MRI of 25 patients, spinal cord MRI of 31 patients, and both head and spinal cord MRI of 19 patients. We observed brain MRI damage in the corpus callosum, basal ganglia, paraventricular region, temporal lobe, medulla oblongata, cerebellar hemisphere, bilateral cerebral hemispheres, and hypothalamus. Spinal MRI lesions involved the cervical spine, thoracic spine, lumbar spine, the entire length of the spinal cord, and spinal meninges. Because not all patients have completed EEG and PET-CT, seven of the patients we collected had deviant EEG results, and two had abnormal PET-CT results. 27 patients were treated solely with pulse dose of intravenous methyl prednisolone (IVMP), 3 patients underwent IVMP therapy in conjunction with antiviral treatment, 3 patients received IVMP therapy along with immunoglobulin therapy, and 7 patients were treated with IVMP in combination with immunosuppressants, including azathioprine and mycophenolate mofetil Table 2.

**Table 2:** Autonomic symptom phenotype, clinical features, treatment and prognosis of 42 patients with glial fibrillary acidic protein IgG positive.

	Cases in this study (3 cases)	Cases in the literature (39 cases)	Total (42 cases)
Female	0	15	15
Male	3	24	27
Autonomic symptoms			
Urinary bladder dysfunction, *n*	3	38	41
Gastrointestinal dysfunction, *n*	0	18	18
Dyshidrosis, *n*	0	2	2
Variability in blood pressure, *n*	0	1	1
Enhancing heart rate variability, *n*	0	4	4
Additional neurological manifestations			
Headache, *n*	1	11	12
Giddy, *n*	0	2	2
Impairment of consciousness, *n*	1	3	4
dyspnea, *n*	1	1	2
Seizure, *n*	0	7	7
Convulsive seizure, *n*	0	1	1
Visual disturbance, *n*	0	11	11
Posterior polar syndrome, *n*	0	6	6
True bulbar paralysis	0	3	3
Paresthesia, *n*	0	14	14
Involuntary movement, *n*	1	2	3
Ataxia, *n*	0	4	4
Limb weakness, *n*	2	28	30
Paresthesia, *n*	0	14	14
Cognitive impairment, *n*	0	3	3
Sleep disorder, *n*	0	3	3
Psychiatric symptom, *n*	0	6	6
Other clinical symptoms			
Fever, *n*	2	18	20
Rash, *n*	0	2	2
Other antibodies			
AQP4	0	11	11
Pathogen			
Epstein–barr virus	1	1	2
MR			
Brain lesion	1	5	6
Spinal lesion	2	10	12
Brain and spinal cord lesions	0	19	19
Electroencephalogram	–	7	7
Cerebrospinal fluid	–	2	2
The accumulation of pressure,n	2	25	27
Leukocytosis, *n*	2	37	39
Protein elevation, *n*	2	27	29
Hypoglycopenia, *n*	0	9	9
Heal			
IVMP therapy alone, *n*	2	25	27
IVMP combined antiviral, *n*	0	3	3
IVMP combined with gamma globulin, *n*	1	2	3
IVMP combined with immunosuppressants, *n*	0	7	7

Correlation analysis of autonomic nerve symptoms: The results showed that Blood pressure variability is related to cerebrospinal fluid pressure and convulsive seizures. Blood pressure variability is related to cerebrospinal fluid pressure and convulsive seizures. Heart rate variability is related to disorders of consciousness. Unusual sweating was associated to age, gender, cerebrospinal fluid protein content and convulsive seizures. Gastrointestinal disorders have associations with gender, sleep disturbances, protein content in the cerebrospinal fluid, and brain MRI lesions. The prognosis of autonomic nerve dysfunction is associated to sleep disorder and cerebrospinal fluid protein content. The higher the cerebrospinal fluid protein level, the worse the prognosis of autonomic nervous system (Tables 3, 4).

**Table 3:** Correlation of blood pressure variability, unusual sweating, gastrointestinal dysfunction, bladder dysfunction and heart rate variability.

Item	Name	Variability in blood pressure(%)		t/χ^2^	*p*	Name	Enhancing heart rate variability(%)		t/χ^2^	*p*	Name	Dyshidrosis(%)		t/χ^2^	*p*	Name	Gastrointestinal dysfunction (%)		t/χ^2^	*p*	Name	Urinary bladder dysfunction(%)		t/χ^2^	*p*
		Non-existence	existence				Non-existence	existence				Non-existence	existence				Non-existence	existence				Non-existence	existence		
sex	Female	15 (100.00)	0 (0.00)	2.456	0.117	Female	15(100.00)	0(0.00)	0.569	0.451	Female	15 (100.00)	0 (0.00)	19.95	0.000**	Female	5 (33.33)	10 (66.67)	5.333	0.020*	Female	1 (6.67)	14 (93.33)	1.844	0.174
	Male	23 (85.19)	4 (14.81)			Male	26(96.30)	1(3.70)			Male	25 (92.59)	2 (7.41)			Male	5 (33.33)	10 (66.67)			male	0 (0.00)	27 (100.00)		
age		45.37 ± 15.68	53.00 ± 20.93	0.900	0.374		45.56 ± 15.92	68.00 ± null	/	/		45.35 ± 16.14	61.00 ± 0.00	−6.133	0.000**		47.00 ± 16.31	44.89 ± 16.20	0.416	0.679		65.00 ± null	45.63 ± 16.02	/	/
Convulsive seizure	Non-existence	38 (92.68)	3 (7.32)	9.732	0.002**	Non-existence	40 (97.56)	1 (2.44)	0.025	0.874	Non-existence	40 (97.56)	1 (2.44)	20.488	0.000**	Non-existence	23 (56.10)	18 (43.90)	0.768	0.381	Non-existence	1 (2.44)	40 (97.56)	0.025	0.874
	existence	0 (0.00)	1 (100.00)			existence	1 (100.00)	0 (0.00)			existence	0 (0.00)	1 (100.00)			existence	1 (100.00)	0 (0.00)			existence	0 (0.00)	1 (100.00)		
Sleep disorder	Non-existence	35 (89.74)	4 (10.26)	0.340	0.560	Non-existence	38 (97.44)	1 (2.56)	0.079	0.779	Non-existence	37 (94.87)	2 (5.13)	0.162	0.688	Non-existence	24 (61.54)	15 (38.46)	4.308	0.038*	Non-existence	1 (2.56)	38 (97.44)	0.079	0.779
	existence	3 (100.00)	0 (0.00)			existence	3 (100.00)	0 (0.00)			existence	3 (100.00)	0 (0.00)			existence	0 (0.00)	3 (100.00)			existence	0 (0.00)	3 (100.00)		
Disturbance of consciousness	Non-existence	35 (92.11)	3 (7.89)	1.229	0.268	Non-existence	38 (100.00)	0 (0.00)	9.732	0.002**	Non-existence	36 (94.74)	2 (5.26)	0.221	0.638	Non-existence	20 (52.63)	18 (47.37)	1.366	0.243	Non-existence	1 (2.63)	37 (97.37)	0.108	0.743
	existence	3 (75.00)	1 (25.00)			existence	3 (75.00)	1 (25.00)			existence	4 (100.00)	0 (0.00)			existence	4 (100.00)	0 (0.00)			existence	0 (0.00)	4 (100.00)		
Head MRI lesion	Non-existence	15 (100.00)	0 (0.00)	2.667	0.102	Non-existence	15 (100.00)	0 (0.00)	0.615	0.433	Non-existence	15 (100.00)	0 (0.00)	1.263	0.261	Non-existence	5 (33.33)	10 (66.67)	4.552	0.033*	Non-existence	0 (0.00)	15 (100.00)	0.615	0.433
	existence	21 (84.00)	4 (16.00)			existence	24 (96.00)	1 (4.00)			existence	23(92.00)	2(8.00)			existence	17 (68.00)	8 (32.00)			existence	1 (4.00)	24 (96.00)		
Spinal cord MRI focus	Non-existence	7 (77.78)	2 (22.22)	1.928	0.165	Non-existence	9 (100.00)	0 (0.00)	0.298	0.585	Non-existence	8(88.89)	1(11.11)	0.913	0.339	Non-existence	6 (66.67)	3 (33.33)	0.639	0.424	Non-existence	0 (0.00)	9 (100.00)	0.298	0.585
	existence	29 (93.55)	2 (6.45)			existence	30 (96.77)	1 (3.23)			existence	30 (96.77)	1 (3.23)			existence	16 (51.61)	15 (48.39)			existence	1 (3.23)	30 (96.77)		
Cerebrospinal fluid pressure		195.53 ± 59.21	130.00 ± 16.33	2.182	0.035*		190.24 ± 60.10	150.00 ± null	/	/		96.83 ± 138.70	20.50 ± 6.36	0.769	0.446		186.38 ± 71.52	193.17 ± 40.54	−0.389	0.699		180.00 ± null	189.51 ± 60.41	/	/
Cerebrospinal fluid white blood cells( × 106 /L)		96.53 ± 141.99	61.50 ± 60.80	0.484	0.631		91.83 ± 137.68	149.00 ± null	/	/		96.83 ± 138.70	20.50 ± 6.36	0.769	0.446		112.00 ± 170.54	68.11 ± 65.39	1.034	0.307		19.00 ± null	95.00 ± 137.46	/	/
Cerebrospinal fluid protein(mg/L)		0.84 ± 0.56	1.00 ± 1.14	0.287	0.792		0.82 ± 0.57	2.36 ± null	/	/		0.89 ± 0.60	0.06 ± 0.01	8.667	0.000**		1.06 ± 0.64	0.57 ± 0.47	2.731	0.009**		0.60 ± null	0.86 ± 0.62	/	/
Cerebrospinal fluid sugar(mmol/L)		3.04 ± 0.76	3.18 ± 0.22	−0.363	0.719		3.06 ± 0.73	3.02 ± null	/	/		3.05 ± 0.74	3.11 ± 0.13	−0.106	0.916		3.21 ± 0.77	2.85 ± 0.61	1.655	0.106		3.30 ± null	3.05 ± 0.73	/	/

* Indicates a statistically significant value.

**Table 4:** Binary logistic regression analysis of the correlation of gastrointestinal dysfunction.

Item	Regression coefficient	Standard error	*z*	Wald χ^2^	*p*	OR	OR 95% CI
Cerebrospinal fluid protein(mg/L)	−1.142	0.712	−1.604	2.574	0.109	0.319	0.079 ~ 1.288
sex	−1.535	0.756	−2.030	4.120	0.042*	0.216	0.049 ~ 0.949
Sleep disorder	18.784	6504.251	0.003	0.000	0.998	143.579	0.000 ~ null
intercept	2.948	1.346	2.190	4.798	0.028	19.063	1.364 ~ 266.466

* Indicates a statistically significant value.

The correlation analysis demonstrated amelioration of autonomic nervous symptoms in the patients across our three cases. We documented autonomic outcomes for 25 patients in the literature, with 16 patients lost to follow-up. We used the independent sample T-test to examine the association between the prognosis of autonomic nervous symptoms and the findings of laboratory studies. The results indicated a significant association between the prognosis of autonomic nervous symptoms and cerebrospinal fluid protein (*p* = 0.001). We use The Chi-square test to investigate the association between autonomic symptoms, other clinical symptoms, and imaging indicators in patients. The findings indicated a significant association between the prognosis of autonomic symptoms and sleep disorders (*p* = 0.043). Patients with sleep disorders have a poor prognosis of the autonomic nervous system, while those with higher cerebrospinal fluid protein content have an even worse prognosis. We included sleep disorders and cerebrospinal fluid protein levels in binary logistic regression analysis to investigate their correlation with the prognosis of the autonomic nervous system. However, no significant correlation was observed (Table 5).

**Table 5:** Correlation of autonomic nervous system prognosis.

	Prognosis of autonomic symptoms (mean ± standard deviation)		*t*	*p*	item	name	Prognosis of autonomic symptoms(%)		*χ* ^2^	*p*
	Good Prognosis (*n* = 22)	Bad Prognosis(*n* = 6)					Good Prognosis (*n* = 22)	Bad Prognosis(*n* = 6)		
Age	47.14 ± 17.82	55.33 ± 16.49	−1.013	0.320	Sleep disorder	existence	21 (84.00)	4 (16.00)	4.084	0.043*
Cerebrospinal fluid pressure	199.77 ± 75.63	152.17 ± 40.10	1.472	0.153						
Cerebrospinal fluid white blood cells( × 106 /L)	99.23 ± 98.95	61.33 ± 86.37	0.851	0.402		Non-existence	1 (33.33)	2 (66.67)		
Cerebrospinal fluid protein(mg/L)	1.07 ± 0.69	0.32 ± 0.27	4.071	0.001**						

* Indicates a statistically significant value.

## 4 Discussion

Autoimmune diseases can impact the autonomic nervous system (ANS) independently or as part of a multifocal involvement. Injury to the sympathetic, parasympathetic, intestinal ganglion, autonomic, or central autonomic nerve pathways can result in autonomic dysfunction, whether separately or in combination. The presence of autonomic dysfunction is a characteristic feature commonly observed in various neurodegenerative diseases such as Parkinson’s disease and multiple system atrophy (4). Autonomic symptoms encompass dysfunctions in gastrointestinal motility, urinary bladder control, and cardiovascular regulation, including blood pressure and heart rate variability, sexual response, thermoregulation, and pupillary function (6). Since 2020, numerous studies and cases have reported autonomic nervous system involvement in GFAP-A. Our study included 42 GFAP-A cases to explore the pathogenesis of autonomic symptoms and their correlation with patient prognosis.

### 4.1 Urinary bladder dysfunction

The autonomic nervous system plays a significant role in regulating the micturition cycle, with the parasympathetic nervous system being crucial during the voiding phase and the sympathetic nervous system playing an essential role during storage and filling (24). The clinical features of GFAP/AQP4-IgG double-positive myelitis include urinary retention and sensory disturbances. At the same time, spinal cord MRI typically reveals extensive lesions spanning more than three spinal segments (25). However, in our case 1, the patient initially presented with dysuria as the sole symptom and sought treatment at the urology department. Despite undergoing two prostate operations, there was no significant improvement in the patient’s urinary symptoms. Following the second prostate operation, the patient developed clinical symptoms of urinary incontinence and subsequently experienced weakness in both lower limbs. In the early stages of GFAP-A, if autonomic nervous dysfunction is the primary symptom, atypical and incomplete symptoms may lead to underdiagnosis and misdiagnosis by clinicians.

### 4.2 Gastrointestinal disorders

Three branches of the autonomic nervous system regulate the movement of the gastrointestinal tract, namely the enteric, parasympathetic, and sympathetic nervous systems (26). In 2020, a 65-year-old man in Japan was diagnosed with autoimmune GFAP-A, presenting clinical symptoms of dyskinesia and autonomic dysfunction, such as urinary difficulty and constipation. Magnetic resonance imaging revealed longitudinal extensive myelopathy (LESCL) from the cervical to the thoracic spine, and treatment with corticosteroids and intravenous immunoglobulin led to clinical improvement (13). Our research found that gastrointestinal dysfunction in GFAP-A patients was associated with the protein content of CSF, gender, brain MRI lesions, and sleep disorders. Gender as an independent factor linked to gastrointestinal dysfunction in GFAP-A. Research has indicated intestinal glial cells are abundant in glial fibrillary acidic protein (GFAP), which is related to 100 glial intermediate filaments. GFAP is to be restricted to astrocytes within the central nervous system (27). Further research is needed to investigate the potential relationship between gastrointestinal autonomic symptoms and GFAP-A.

### 4.3 Cardiac sympathetic nerve dysfunction

Cardiac sympathetic dysfunction is also present in patients with GFAP-A. In 2023, a 68-year-old GFAP-A patient in China gradually developed heart rate variability and non-dipper circadian rhythm of blood pressure during hospitalization after intravenous immunoglobulin and corticosteroid treatment. The patient’s symptoms have significantly improved. This case broadens the range of expected symptoms of GFAP-A syndrome, as it manifests as heart rate variability and blood pressure variability (11). Dysregulation of blood pressure and heart rate may be correlated with adverse outcomes in patients with severe anti-NMDAR encephalitis. At the same time, the duration of NICU stay and mechanical ventilation is prolonged in patients with paroxysmal sympathetic hyperexcitation (28). The involvement of GFAP-A in cardiac autonomic function remains poorly understood. It has been documented that antibodies targeting NMDA receptors can disrupt sympathetic circuits by modulating vagus nerve output in the brain stem and regulating sympathetic nerve output in the hypothalamus and spinal cord, ultimately leading to cardiac sympathetic dysfunction in anti-NMDAR encephalitis (29). The potential impact of GFAP antibodies on heart rate stability through cytotoxic T cell-mediated autoimmune responses targeting sympathetic circuits requires further investigation.

### 4.4 Excessive sweating

The CNS and ANS regulate the secretion of sweat. Research has indicated that lesions in the anterior region of the hypothalamus result in hyperthermia, while lesions in the posterior region lead to hypothermia, often accompanied by disruptions in the sweating mechanism (30). Our study suggests abnormal sweating is associated with age, sex, cerebrospinal fluid protein content, and fierce attack. Further research is required to investigate the relationship between untoward GFAP-A sweating and brain lesions responsible for regulating body temperature and thermoregulatory effector responses or the impact of GFAP-A antibodies on neurotransmitter release in peripheral ganglia.

Studies have indicated that most patients exhibited expected EEG results in GFAP-A patients with lesions only on spinal cord MRI but no specific changes on head MRI. At the same time, a small portion showed mild abnormalities (31). However, based on our case studies and literature, there are relatively few patients who have completed EEG and PET-CT examinations. Our findings indicate that the prognosis of autonomic symptoms may be linked to sleep disorders. We conducted binary logistic regression, including sleep disorders and cerebrospinal fluid protein content, to investigate the independent association with autonomic nervous system prognosis but found no significant correlation. The small sample size may have contributed to the lack of statistical significance in the influence of factors. Secondly, multiple factors such as genetics, environment, and lifestyle can affect the improvement of GFAP-A autonomic symptoms, which statistical analysis cannot explain. Furthermore, unidentified cellular, molecular, and biological mechanisms may impact the prognosis of the autonomic nervous system, yet we have not fully comprehended their mode of action. The impairment of astrocytes within the autonomic neural network could potentially give rise to autonomic clinical symptoms in GFAP-A. Further pathological investigations and the establishment of animal models are imperative for elucidating the pathogenesis of astrocyte dysfunction and its correlation with autonomic involvement in GFAP-IGG-associated autoimmune astrocytoma.

The latest research elucidates the neuropathology and pathogenesis of GFAP-A. Their histopathological studies indicate that GFAP autoimmune response is mediated by cytotoxic T cells, and C4D deposits on astrocytes can represent the cause or result of astrocyte reactivity (32). In 2024, a systematic review of the clinical and neuroimaging phenotypes of GFAP-A suggests that GFAP-A can present with a range of neuroimaging and clinical findings. A high clinical awareness of GFAP-A is therefore necessary in the diagnostic workup of patients with noninfectious encephalitis and meningeal features, and prompt testing of GFAP-IgG is recommended. Neuroradiological findings of perivascular contrast enhancement, deep grey matter involvement, and longitudinally extensive myelitis may be indicative of GFAP-A. Detection of coexisting autoantibodies and/or concomitant malignancy are important factors to consider in the diagnostic workup of suspected cases. Future studies should elaborate on the described clinical, laboratory, and imaging features, explore the pediatric panorama of GFAP-A, and evaluate the role of advanced MRI in the diagnostics and management of GFAP-A (33). A case of GFAP-A was reported in 2024, and due to his clinical symptoms, cerebrospinal fluid analysis, initial normal brain imaging, and negative serum autoimmune encephalopathy group, his initial diagnosis was presumed to be viral meningoencephalitis. Through this case, the researchers highlighted the importance of appropriately detecting serum and cerebrospinal fluid autoantibodies during the progression of autoimmune encephalitis (34). There are studies showing that first-line immunotherapy is effective for most patients with acute autoimmune GFAP -A. Most patients may experience relapse when taking oral steroids, and relapse is usually accompanied by worsening of previous symptoms. A small number of patients may also develop new symptoms, and they have found that children have better short-term prognosis (35).

## Data Availability

The original contributions presented in the study are included in the article/supplementary material, further inquiries can be directed to the corresponding authors.
